# Characterization of the Aroma Profiles of Guangdong Black Teas Using Non-Targeted Metabolomics

**DOI:** 10.3390/foods12071560

**Published:** 2023-04-06

**Authors:** Qiushuang Wang, Dandan Qin, Xiaohui Jiang, Kaixing Fang, Bo Li, Qing Wang, Chendong Pan, Erdong Ni, Hongjian Li, Dong Chen, Hualing Wu

**Affiliations:** Tea Research Institute, Guangdong Academy of Agricultural Sciences, Guangdong Provincial Key Laboratory of Tea Plant Resources Innovation and Utilization, Guangzhou 510640, China

**Keywords:** black teas, volatile flavor compounds, metabolomics

## Abstract

Guangdong black teas have diverse flavors and aromas. To explore the molecular basis of these aromas, we extracted and analyzed the volatile flavor compounds of 31 black tea samples from 7 districts (Yingde, Luokeng, Renhua, Meizhou, Chaozhou, Lianshan, and Heyuan) in Guangdong Province with headspace solid-phase microextraction (HS-SPME) coupled with gas chromatography–mass spectrometry (GC–MS). Then, 135 volatile flavor compounds (VFCs) were identified and grouped into 12 classes according to their chemical structure. Notably, alcohols accounted for 31.40–44.43% of total VFCs. The score plot of supervised partial least squares-discriminant analysis (PLS-DA) revealed good discrimination for most black tea samples. Additionally, 64 compounds with variable importance in projection > 1.0 were identified as differential odorants. Through an odor activity value analysis, eight volatile compounds were identified as the key active differential VFCs: linalool, methyl salicylate, phenylethyl alcohol, *p*-cresol, 3-methyl-butanoic acid, geraniol, benzaldehyde, and benzeneacetaldehyde. Thus, benzeneacetaldehyde and linalool in YJ-Yingde samples, benzaldehyde in Luokeng samples with an almond-like aroma, phenylethyl alcohol in the Heyuan samples, and *p*-cresol and 3-methyl-butanoic acid in the Chaozhou samples were the key volatile flavor compounds that could differentiate local black teas from other black teas. These findings will enrich the research in tea aroma chemistry and provide a method for identifying the origins of Guangdong black teas.

## 1. Introduction

Given its unique and fragrant aroma, black tea is the most popular tea in international tea markets, accounting for more than 78% of total tea consumption globally. In addition, the unique floral, sweet, honey-like, and fruity aromas of black teas are considered intoxicating. These multitudinous aromas are attributed to the high quantities of volatile flavor compounds (VFCs) in black teas. To date, more than 700 VFCs have been identified using non-targeted metabolomics based on gas chromatography–mass spectrometry (GC–MS), comprehensive two-dimensional gas chromatography coupled with time-of-flight mass spectrometry (GC×GC–TOFMS), and gas chromatography–olfactometry/gas chromatography–mass spectrometry (GC–O/GC–MS) [1,2]. However, few identified VFCs contribute to the overall aromatic characteristics in teas. These compounds are known as key odorants or key active aromatic compounds. In recent years, research on the key odorants in teas has attracted considerable attention [3,4,5,6].

Diverse aroma substances in black teas have been found in tea science research. Among the key odorants in Guangdong Yingde black tea brewed at different temperatures, 16 compounds, including linalool, methyl salicylate, *β*-ionone, *β*-damascone, nonanal, ethyl hexanoate, dimethyl sulfide, and cedrol, have been found to contribute significantly to the total aroma [7]. In addition, the relative concentrations of 20 key odorants, including benzeneacetaldehyde, *β*-damascenone, and linalool, significantly differ among the Yingde black teas with different harvest seasons [8]. Furthermore, 19 key odorants identified can differentiate Assam, Keemun, and Ceylon black teas because of their aromatic activities [9]. The characteristic odorous compounds are (*E*)-2-octenal in Assam black tea, benzeneacetaldehyde in Keemun black tea, and methyl salicylate in Ceylon black tea [9]. A recent study showed that alcohols such as linalool and geraniol contribute to the floral or fruity notes in Keemun black tea during the sun withering process [10]. Besides black teas, other teas, such as green tea, oolong tea, white tea, and dark tea, have been similarly studied [11,12,13,14].

Owing to the complexity of the abundant aromatic substances identified using GC–MS, non-targeted metabolomic analysis methods have been widely applied to tea aroma studies. These methods include principal component analysis (PCA), partial least squares-discriminant analysis (PLS-DA), and hierarchical cluster analysis (HCA) [15]. These analyses can help differentiate key chemical components among different sample groups and investigate the intrinsic connections between chemical components and samples [16].

Guangdong Province is a major Congou black-tea-producing area in China. Among the Congou black teas from Guangdong, Yinghong No. 9 is the most widely known and representative large-leaf black tea, and it is as famous as Keemun black tea from Anhui Province, Darjeeling from India, and Ceylon from Sri Lanka. It typically has a sweet, orchid-like aroma. Furthermore, recently bred black tea varieties from Guangdong have diverse scents, such as almond-like, medicinal, and honey-like aromas. However, these aromas have not been systematically studied, and the key aroma substances contributing to them remain unclear. In the present research, 31 typical black teas from 7 different producing areas in Guangdong Province were comprehensively analyzed using non-targeted metabolomics. The key VFCs were determined and screened using headspace solid-phase microextraction (HS-SPME) coupled with GC–MS. The purpose of this research was to identify the key aroma indicators that can distinguish Guangdong black tea samples. These results can enrich tea aroma research and provide a method for identifying the origins of Guangdong black teas.

## 2. Materials and Methods

### 2.1. Black Tea Samples

**Figure 1:**
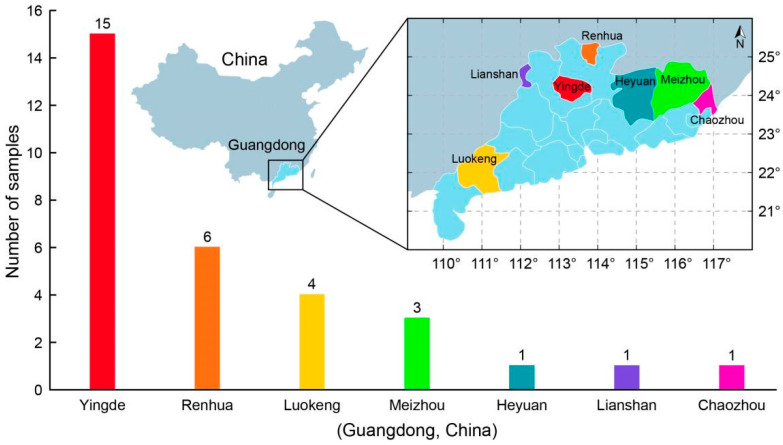
Black tea samples in Guangdong Province, China.

A total of 31 black tea (*Camellia sinensis* L.) samples were obtained from 7 different districts of Guangdong Province in 2019 by experienced tea tasters: 15 Yingde black teas (samples 1 to 15), 4 Luokeng black teas (samples 16 to 19), 6 Renhua black teas (samples 20 to 25), 3 Meizhou black teas (samples 26 to 28), and 1 each of Heyuan (sample 29), Lianshan (sample 30), and Chaozhou (sample 31) black teas (Figure 1). Prior to sample extraction, tea samples were kept in a 4 °C refrigerator for further analysis.

### 2.2. Aroma Evaluation

All black teas were evaluated according to the Standard Methodology of the People’s Republic of China, ‘Sensory Evaluation of Tea’ (GB/T 23776-2018) [17] by five trained panelists (three females and two males, 40–52 years of age). This evaluation was conducted at approximately 25 °C in our lab, and the primary focus was the aromas of the black teas. The five panelists determined the sensory aromas and provided their comments.

### 2.3. Extraction and Identification of the VFCs

The extraction of the VFCs and the GC–MS identification procedures employed were identical to those of our previous study [18]. Briefly, a ground black tea sample (1.5 g) and ethyl decanoate (20 µL, 0.02 mg/mL), as an internal standard, were transferred to a SPME bottle. The automatic sampler extracted the VFCs in these samples. After that, the VFCs extracted were analyzed on a GC–MS system. All samples were performed in six repetitions, which was also conducted for the quality control samples.

Data processing was according to MS-DIAL software [18]. Different from the traditional data-dependent MS/MS acquisition method, the data independent acquisition (DIA) method can acquire all the fragments of ions in all precursors simultaneously therefore, DIA can increase the coverage of observable molecules and reduce the identification of false negatives. MS-DIAL is an advanced data processing method based on DIA to identify and quantify of the small molecules via MS deconvolution. In this study, during data processing, the raw data of GC/MS (.D format) were converted to .abf format by AnalysisBaseFileConverter software for a quick search of volatile compounds, which were then imported into the MS-DIAL software for pre-processing. The ion peaks of precursors were effectively discovered by analyzing the two consecutive data axes; and the ‘model peaks’ in the chromatography were extracted by the MS2Dec algorithm to remove the background noise. Finally, the volatile compounds were identified according to the retention time, precise molecular mass, mass spectra, and the matching similarity in the public database, the NIST library (accessed on June 2014). The relative concentration of the VFCs was semi-quantified and calculated according to the content and peak area of the ethyl decanoate as follows [18]:

(1) $$Relative\;concentration\left(ng/g\right)=\frac{peak\;area\;of\;each\;compound\times mss\;of\;ethyl\;decanonate}{peak\;area\;of\;ethyl\;decanonate\times dry\;mass\;of\;sample}$$

Additionally, the concentration of the VFC (ng/g) shown in the study referred to the relative concentration calculated based on the internal standard ethyl decanoate.

### 2.4. The Odor Activity Value (OAV) Calculation

OAV is a parameter applied in aroma research to determine the key active flavor compounds and understand their effects on the overall aroma in teas [19]. VFCs with an OAV greater than 1 are considered key active volatile compounds. In this research, the OAV was calculated according to the method of Wang et al. [19], and the odor threshold (OT) used in this study was the OT in air [20].

### 2.5. Multivariate Analysis

A multivariate analysis was conducted to identify the characteristic volatile compounds in the different black tea groups. In this research, supervised PLS-DA was conducted using SIMCA (version 14.0, Umetrics, Umea, Sweden). Hotelling’s *T*^2^ region, shown as an ellipse in the score plots of the models, was defined as the 95% confidence interval of the modeled variation. The cross-validation test (200 random permutations) was used to measure overfitting of the former model. From the cross-validation test, the variable importance in the projection (VIP) was generated, and VFCs with VIP > 1 were identified as potential characteristic compounds [13,14]. The significant differences in VFCs between the different black tea samples were analyzed using one-way analysis of variance (ANOVA) (GraphPad Prism 8.0.2, GraphPad Software Co., San Diego, CA, USA,). Differences were considered significant at *p* < 0.05.

## 3. Results and Discussion

### 3.1. Aroma Evaluation

Each black tea sample was evaluated and described by the five panelists. The results showed that all the black teas presented sweet scents (Table S1), similarly to the teas reported in the literature [21,22,23]. Sweet odorants were found in all samples, which indicates that this sweet characteristic is shared among the black teas from Guangdong, which is consistent with the general aroma of black teas in the current standards [18]. High-quality black teas also have floral aromas, and some black tea samples have specific medicinal, wood-like, musk-like, or almond-like aromas.

Black teas in Yingde had two types of aromas: a sweet, orchid-like aroma and a sweet aroma with typical floral notes. The Luokeng black teas had two types of aromas: a strong almond-sweet aroma and a woody-sweet aroma. Most Renhua black teas had intoxicating medicinal and sweet aromas. Meizhou black teas presented sweet and flower-like odors. Heyuan black tea exhibited a sweet aroma with honey-like notes, and Chaozhou black tea exhibited sweet, floral, and medicinal aromas. Lianshan black tea presented a medicinal and sweet aroma. Floral and sweet aromas are typically attributed to geraniol, *β*-ionone, linalool, phenylethyl alcohol, benzeneacetaldehyde, and *β*-damascenone [22]. Mint- or peppermint-like aromas, exemplified by teas from Renhua, are attributed to methyl salicylate or citral [18,23].

### 3.2. Identification of Volatile Compounds

The VFCs in each black tea sample were analyzed using GC–MS and then tentatively identified using the NIST library (98 L), retention time, and retention index, and they were positively identified using authentic compounds, if available. During identification and quantification, the peaks associated with noise, byproducts produced by column bleeding, halogen compounds, and impurities were removed from the final compounds list. The peaks of the same VFC were then merged.

**Figure 2:**
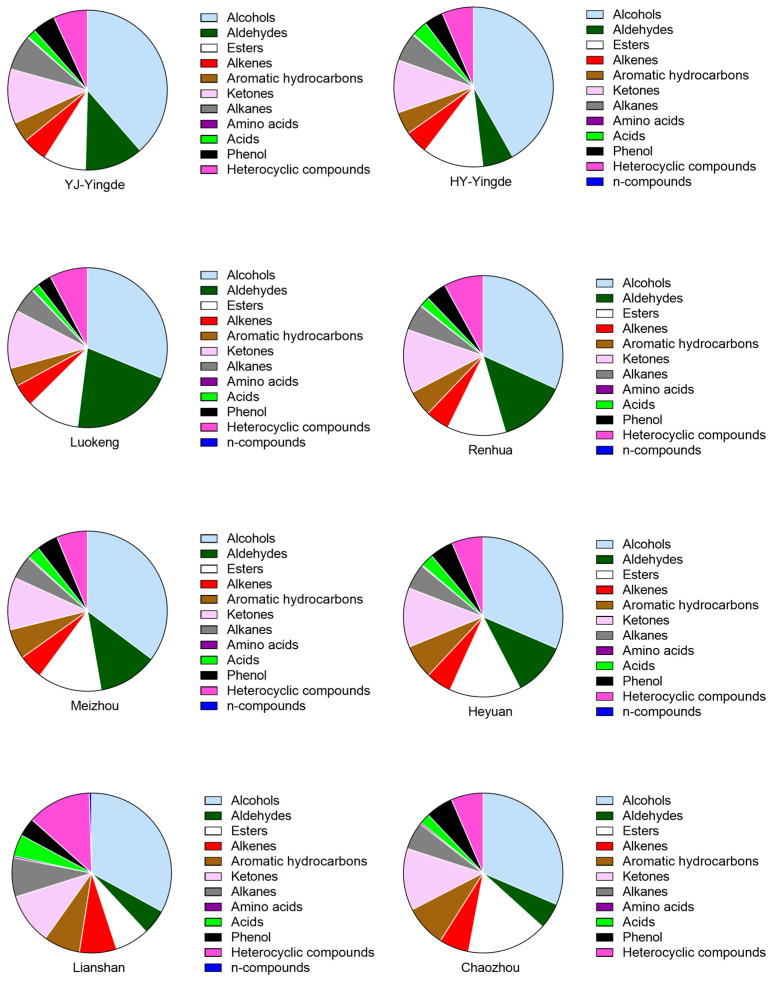
Proportions of the 12 identified groups of VFCs in black tea samples from 7 districts in Guangdong Province.

In total, 135 VFCs were identified and categorized into 12 groups according to their chemical structures. There were 24 alcohols, 25 heterocyclic compounds, 9 aldehydes, 17 ketones, 10 alkenes, 6 acids, 9 aromatic hydrocarbons, 17 esters, 9 alkanes, 3 amino acids, 5 phenols, and 1 N-compound, as shown in Table S2. The proportions of the 12 identified groups are shown in Figure 2. Notably, alcohols made up the highest proportion among the samples in the seven regions, accounting for 31.40–44.43% of total VFCs, so they constituted the primary VFC group. In particular, the relative content of alcohols in the Yingde black teas (average of 40.20% of total VFCs) were significantly higher than that of samples in other districts (*p* < 0.05). Among the alcohols detected, linalool existed in the highest average relative concentration (2066.04 ng/g, calculated based on ethyl decanoate) in the YJ-Yingde black teas, followed by geraniol in the Meizhou black teas (1891.58 ng/g) and phenylethyl alcohol (1621.01 ng/g) in the Heyuan black tea sample (Table S2).

Although the HY-Yingde black teas and YJ-Yingde black teas were from Yingde City, they exhibited different aroma profiles, especially in the proportions of aldehydes and acids. For example, the relative content of aldehydes in the YJ-Yingde black teas was nearly double that in the HY-Yingde black teas, accounting for 11.85% of total VFCs in the former. In contrast, the relative content of acids in the HY-Yingde black teas was nearly twice that in the YJ-Yingde black teas. The YJ-Yingde and HY-Yingde black teas were divided into different groups because they came from different tea varieties, which could have led to the detection of different aroma characteristics. YJ-Yingde black teas were processed from *Camellia sinensis var. assamica* (Masters) Kitamura cv. Yinghong No. 9, a predominant tea variety of Yingde City, Guangdong Province, which was bred from a Yunnan large-leaf tea and is suitable for producing black and white teas. HY-Yingde black teas were made from *Camellia sinensis* (L.) O. *Kitamura* cv. Hongyan No. 12, which was screened from a natural hybrid offspring of the *Tieguanyin* variety planted in Yingde district; it is a good tea variety with wide applicability in making green, black, and oolong teas. Different tea varieties should possess diverse aroma profiles, which is supported by the results of the aroma evaluation (Table S1). YJ-Yingde black teas exhibited sweet and orchid aromas, and the HY-Yingde black teas had sweet, floral aromas.

In addition to abundant alcohols, esters, aldehydes, and ketones were also present in large proportions in the investigated black teas. Among the 12 identified groups of VFCs, esters accounted for 6.89%–16.29% of total aromatics. Among the esters, menthyl salicylate was found in the highest proportion. This compound contributes to the fresh, peppermint-like aroma of teas [21,22]. The highest relative concentration of menthyl salicylate was 996.06 ng/g, which was associated with the black teas from HY-Yingde. Menthyl salicylate has been detected in various teas and is a key volatile compound [4,5,6]. The fine sharpness of black tea aromas was attributed to menthyl salicylate, as shown in the results of the aroma evaluation (Table S1).

Nine aldehydes were detected, including benzaldehyde, benzeneacetaldehyde, (*E*, *E*)-2, 4-heptadienal, 2, 5-dimethyl-benzaldehyde, hexanal, and citral. They accounted for 5.24–20.79% of the total aromatics. Most aldehydes have low odor thresholds and pleasant, fresh aromas [22]. Among the aldehydes detected, benzaldehyde was present in the highest relative concentration in Luokeng black teas (2619.61 ng/g in one sample and 1824.31 ng/g in another sample) (Table S2). The second most abundant aldehyde was benzeneacetaldehyde, which was present in the highest relative concentration in YJ-Yingde black teas, reaching 377.02 ng/g. Benzeneacetaldehyde is found in many black teas and significantly contributes to their sweet aromas [9].

Ketones accounted for 8.92–12.90% of total VFCs. Among the ketones detected, *β*-ionone existed in the highest relative concentration in Meizhou black teas (278.91 ng/g). *β*-Ionone is also an important volatile compound in black and green teas that contributes to the sweet, floral note of tea aromas [5,18].

The acids identified in this study, including 3-methyl-butanoic acid, (E)-benzeneacetic acid, and hexanoic acid, accounted for 1.43–4.4% of total VFCs. As a result of fermentation, acids are often detected in black teas, which affect their overall quality. For example, Indian crush, tear, curl (CTC) black teas, which contain volatile acids such as hexanoic acid and pentanoic acid in high relative concentrations, are generally heavily fermented [18].

### 3.3. Multivariate Statistical Analysis

**Figure 3:**
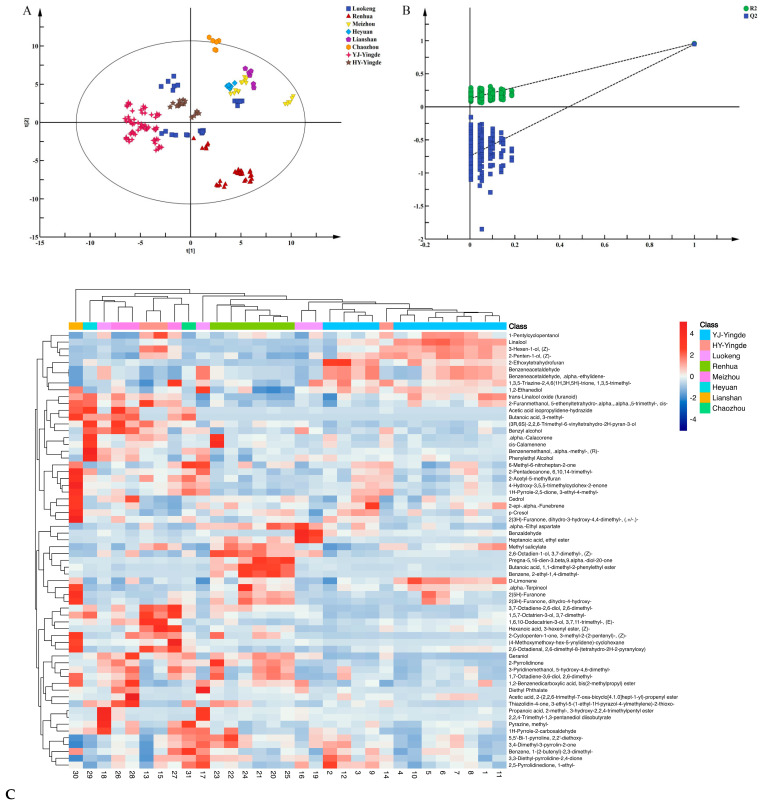
Profiles of VFCs in black teas from seven different areas in Guangdong Province. (**A**) PLS−DA score plot for VFCs in the black tea samples (*R^2^X* = 0.787, *R^2^Y* = 0.944, *Q^2^* = 0.926). (**B**) Cross-validation plot with 200 calculations by permutation tests (*R^2^* = 0.0, 0.129, *Q^2^* = 0.0, −0.744). (**C**) Heat map and hierarchical clustering of the 64 differential substances (VIP > 1 in PLS-DA). The red areas (intensity range of 0−4) present higher relative abundances of the compounds, while the blue areas show lower relative abundances (intensity range of 0 to −4).

A total of 135 VFCs in black teas from the 7 different production areas were analyzed via supervised PLS-DA, and potential chemical markers were identified. The score plot is shown in Figure 3. From Figure 3A, the interpretation rate *R^2^Y* (0.944) and prediction rate *Q^2^* (0.926) show that the PLS-DA model could explain and predict the differences between groups of black tea samples, which indicates that the PLS-DA model is reliable. Duplicate data from the same sample were clustered together, indicating the repeatability of the experiment. Good discrimination was found for most black tea samples from the different production areas in Guangdong. However, samples from the same production region, such as Luokeng, Meizhou, and Renhua, were scattered in different areas of the plot. Samples from Luokeng were dispersed among three different groups, and they were from different wild tea communities separated by high mountains and thick forests [22]. Each black tea was made from a single tea community, resulting in different aroma characteristics. For example, Luokeng black tea samples 16 and 19 had almond-like characteristics primarily attributed to benzaldehyde [22], while samples 17 and 18 displayed a combination of woody and sweet aromas.

Black tea samples from Meizhou were distributed among two groups. Samples 26 and 27 were from Wuhua County, Meizhou City, and were made from Dancong, a famous oolong tea variety from Guangdong [24,25]. Dancong tea originates from Fenghuang Mountain in Chaozhou City, in the east of Guangdong Province, China [24,25], and it can be processed into oolong and black teas. Dancong teas are widely known for their special floral, honey-like, and fruity flavors, including orchid (Zhilan in Chinese), honey-orchid (Milan in Chinese), gardenia (Huangzhi in Chinese), jasmine (Moli in Chinese), and grapefruit (Youhua in Chinese) [24,25]. Sample 28 was from Dapu Town, Meizhou City, and it was processed from locally dominant Meizhan, a medium-leaf variety from Fujian Province that can be processed into black and oolong teas. Because these samples were from different regions (Dancong or Meizhan variety), they exhibited distinct aroma profiles scattered among different groups.

The Renhua black tea samples were clustered in one large group, which included three subgroups, primarily because these six samples (samples 20–25) were from different tea varieties, although they all belonged to the same tea variant, Camellia sinensis var. pubilimba Chang. The three samples of Danxia No. 8 (samples 20, 21, and 25) were clustered, as shown in the lower-right corner of Figure 3A, next to the sample of Danxia No. 4 (sample 23) and below the sample of Danxia No. 5 (sample 24), indicating that different varieties from the same community have different aroma profiles (Table S1). From the sensory evaluation (Table S1), Danxia No. 8 had a special orchid-like aroma, and Danxia No. 4 had a rose-like scent. However, Danxia No. 5 had a distinctive Chinese fir-like aroma. The Lianshan black tea samples clustered together, and the Heyuan black tea samples were close to them (Figure 3A). Lianshan black tea had medicinal aromas, while Heyuan black tea exhibited sweet and honey aromas (Table S1).

A cross-validation test (200 random permutations) was performed using SIMCA (Figure 3B). The results show that the PLS-DA model was reliable (R^2^ = 0.129, Q^2^ = −0.744). Generally, all Q^2^ (blue points) values on the left should be lower than the original point on the right; and the regression line of Q^2^ should intersect with the vertical axis. From Figure 3B, the parameters obtained from the cross-validation test met the above requirements, which indicated that the model of the PLS-DA is not over-fitted and the result of the grouping is reliable. In total, 64 differential compounds with variable importance in projection (VIP) > 1.0 were determined as key compounds with significantly different relative concentrations among the black teas from seven different areas in Guangdong. A heat map of the components with VIP > 1.0 is shown in Figure 3C. Samples with identical origins were primarily clustered together, for example, samples from YJ-Yingde or Renhua, which agrees with the PLS-DA results (Figure 3A). Specifically, samples 1–12 (YJ-Yingde), which were from the same tea variety, Yinghong No. 9, and had similar sweet, orchid-like aromas, were clustered together. They contained linalool, (Z)-2-penten-1-ol, benzeneacetaldehyde, (Z)-3-hexen-1-ol, trans-linalool oxide (furanoid), and D-limonene in high relative concentrations. Similarly, samples 13 and 15, derived from a different tea variety, Hongyan No. 12, were clustered together. They contained 3-methyl-butanoic acid and benzyl alcohol in high relative concentrations. Samples 16 and 19, from Luokeng, were clustered together, and they contained higher relative concentrations of benzaldehyde, heptanoic acid, and ethyl ester than the other samples, as shown in Figure 3C. According to the sensory evaluation, samples 16 and 19 both had almond-like aromas (Table S1). Samples 20–25, from Renhua, were all clustered together because they were derived from the same variety, *Baimao* (*Camellia sinensis var. pubilimba Chang*) from Renhua County [26], and they contained methyl salicylate, benzaldehyde, 2-ethyl-1,4-dimethyl-benzene, butanoic acid, 1, and 1-dimethyl-2-phenylethyl ester in high relative concentrations.

### 3.4. Key Active Compounds Identification of Black Teas in Guangdong

**Figure 4:**
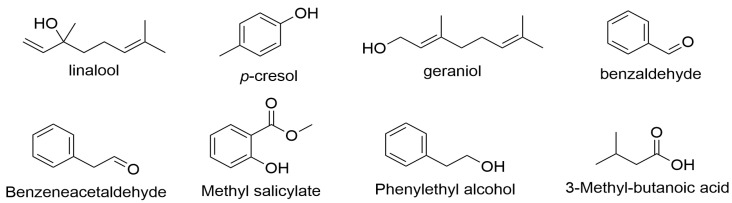
The chemical structures of the eight key active volatile compounds.

The differential VFCs of the black teas in seven districts of Guangdong were investigated. However, it was unclear which substances contributed to the total aromatic characters in these black teas. Therefore, the OAVs of the 64 differential volatile compounds (VIP > 1) were determined using Equation (1) to identify the differential key active odorants. Eight compounds had OAVs > 1 and thus were considered key active differential odorants of Guangdong black tea samples, as shown in Table 1. These compounds were geraniol, methyl salicylate, phenylethyl alcohol, linalool, *p*-cresol, 3-methyl-butanoic acid, benzaldehyde, and benzeneacetaldehyde, all of which played a major role in the overall aroma of the black teas. The chemical structures of the eight key aromatic compounds are shown in Figure 4. Among the eight key active differential odorants, six compounds had OAVs greater than 1 in all of the samples: phenylethyl alcohol, methyl salicylate, *p*-cresol, benzeneacetaldehyde, linalool, and geraniol. However, benzaldehyde had an OAV > 1 only in Luokeng black tea samples, and 3-methyl-butanoic acid had an OAV > 1 only in the Meizhou, Heyuan, Lianshan, and Chaozhou black tea samples. The lower OAVs of benzaldehyde and 3-methyl-butanoic acid may have been caused by the lower relative concentrations in certain samples.

**Figure 5:**
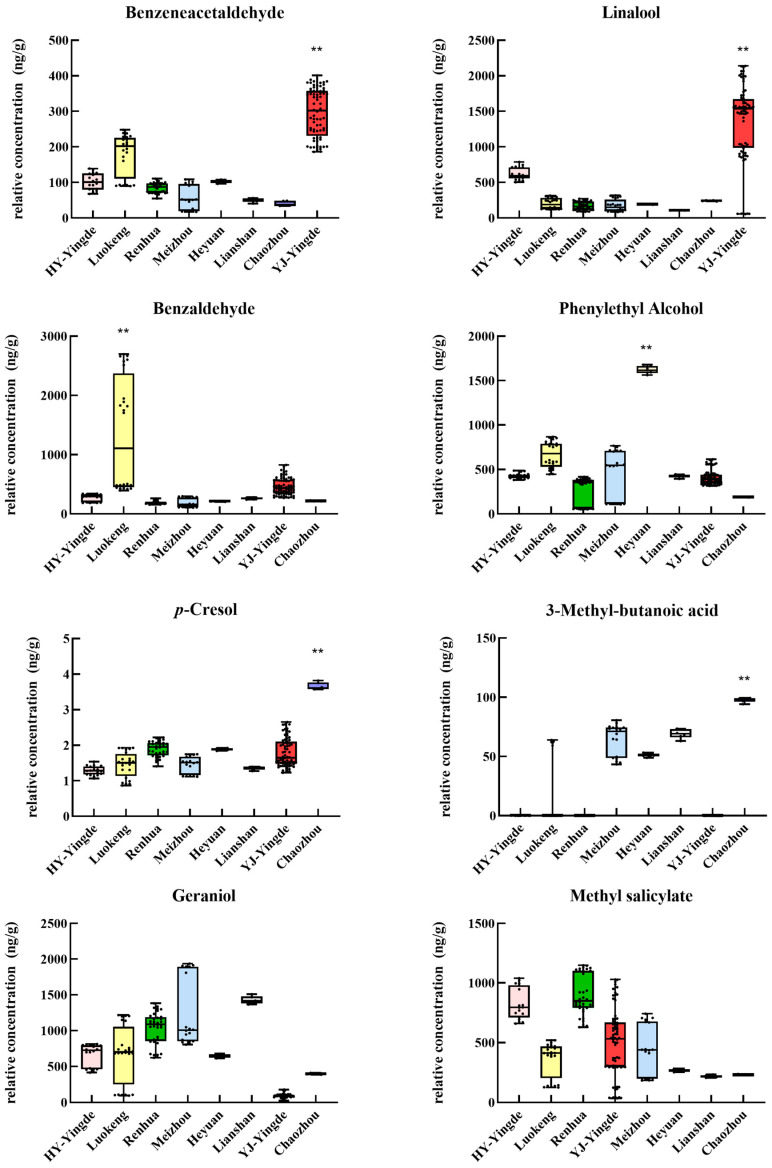
Relative concentration of nine differential VFCs with an OAV greater than 1 in black teas from the seven districts in Guangdong Province. ** indicates that the relative content of the compound in this district is significantly different (higher) than that in other districts (*p* < 0.01). The relative concentration of the nine differential VFCs presented here is calculated according to the concentration of ethyl decanoate.

Figure 5 shows the relative concentrations of the eight key active differential compounds in Guangdong black tea samples. YJ-Yingde black teas had significantly higher relative concentrations and OAVs of benzeneacetaldehyde and linalool than other Guangdong samples. Therefore, they can be considered as markers for distinguishing black teas in YJ-Yingde from other districts in Guangdong, which agrees with the results of previous studies [8,18]. Linalool and benzeneacetaldehyde contribute to the sweet, orchid-like aroma of black teas and can be determined as the key odorants in black teas [9].

It is understood that benzaldehyde contributes to almond-like aromas. The relative concentration of benzaldehyde was significantly different in the Luokeng samples than other Guangdong black tea samples. The Luokeng black tea samples were the only samples containing benzaldehyde with an OAV > 1. Therefore, benzaldehyde may be a key active compound in Luokeng black teas. This result is consistent with that of the aroma evaluation (Table S1), in which the Luokeng samples (samples 16 and 19) had specific almond-like aromas. Our previous study showed that benzaldehyde was relatively stable during the processing of Luokeng black teas, from the fresh tea buds to the dried black teas [22]. Therefore, benzaldehyde can be used as an indicator or marker for identifying black tea varieties with almond-like aromas. Notably, the Luokeng black teas had sweet, woody aromas (samples 17 and 18) and almond-like aromas.

Phenylethyl alcohol is known to contribute to aromas reminiscent of honey, spice, rose, and lilac [9]. Notably, phenylethyl alcohol had the highest relative concentration (1621.01 ng/g) and OAV (10.31) in the Heyuan black tea sample, which is significantly higher than that in the other black tea samples. Thus, phenylethyl alcohol can be used as a discriminator to separate Heyuan black tea from other Guangdong black teas. In the aroma evaluation, the Heyuan black tea exhibited a sweet, honey-like aroma, and this result agrees with the above results. Phenylethyl alcohol is a key aroma substance of honey-like fragrances and is also a key volatile compound in many types of teas, such as oolong teas, black teas, white teas, and green teas [27,28,29].

Menthyl salicylate (peppermint) had the highest relative concentration (1121.95 ng/g) and OAV (4.22) in one sample of Renhua black teas (samples 20–25), significantly different from other samples except HY-Yingde black teas. Menthyl salicylate is present in many types of teas [29,30] and imparts a peppermint note, giving teas a fresh, sharp, easily identifiable aroma. Geraniol (contributing to rose- and geranium-like aromas) had the highest relative concentration (1426 ng/g) in the Lianshan black tea sample, which was significantly different from other samples except Meizhou black tea. A study showed that small-leaf and medium-leaf varieties of tea contained more geraniol than large-leaf tea varieties. Conversely, the latter contained more linalool than the former [18]. In this study, the tea variety of YJ-Yingde black tea was a large-leaf species that contained a higher relative concentration of linalool, while the tea variety of Lianshan black tea was a medium-leaf species containing a higher relative content of geraniol.

*p*-cresol, a phenolic acid, has unique medicinal, phenolic, or smokey characteristics, and it was present in the highest relative concentration (3.66 ng/g) in the Chaozhou black tea sample. 3-Methyl-butanoic acid, contributing to the sour aroma of black teas, was also present in the highest relative concentration (97.79 ng/g) in the Chaozhou black tea. This finding agrees with the unpleasant sour smell obtained in the aroma quality evaluation. One-way ANOVA showed that the Chaozhou black tea sample contained significantly higher relative concentrations of *p*-cresol and 3-methyl-butanoic acid than other Guangdong black teas. Hence, these compounds can separate the Chaozhou sample from other Guangdong samples. In addition, the Chaozhou black tea sample contained linalool, phenylethyl alcohol, geraniol, and benzeneacetaldehyde, and these contributed to the sweet aroma.

## 4. Conclusions

Volatile compounds of 31 black teas originating from 7 different districts in Guangdong, China, were analyzed with GC–MS and non-targeted metabolomics. In total, 135 VFCs were quantified and classified into 12 groups according to chemical structure. The primary VFC group comprised alcohols, which accounted for 31.40–44.43% of total VFCs. The results of supervised PLS-DA indicate that samples from the same production area in Guangdong were clustered together. Furthermore, 64 VFCs (VIP > 1.0) were determined as the differential compounds in these black tea samples. From the OAV analysis, eight volatile compounds—that is, linalool, methyl salicylate, phenylethyl alcohol, *p*-cresol, 3-methyl-butanoic acid, geraniol, benzaldehyde, and benzeneacetaldehyde—were the key active differential compounds. Linalool and benzeneacetaldehyde were potential biomarkers for the sweet, orchid-like aromas of YJ-Yingde black teas, so these volatile compounds can differentiate YJ-Yingde tea samples from other Guangdong samples of black teas. Benzaldehyde was the specific material foundation for the almond scents in Luokeng samples. Phenylethyl alcohol was the key compound for the sweet aromas in Heyuan black tea. *p*-cresol and 3-methyl-butanoic acid were important compounds contributing to the sour aroma in the Chaozhou tea sample. The above key VFCs can be used as discriminators to separate them from black tea produced in other areas of Guangdong. The results of aroma evaluation suggest that the common aroma characteristics in Guangdong black tea were sweet and floral odors, although still other black teas presented minty, green, almond, or honey-like aromas, which agrees with the GC–MS analysis. These results will enrich the aroma chemistry studies of teas and provide a practical method for research to identify the origins of Guangdong black teas.

## Figures and Tables

**Table 1:** OAVs in the eight key VFCs in Guangdong black tea samples.

Name of Odorants	Odor Notes ^a^	YJ-Yingde	HY-Yingde	Luokeng	Renhua	Meizhou	Heyuan	Lianshan	Chaozhou
Linalool	floral, lavender	42.48 *	19.50	6.32	5.31	6.00	6.05	7.56	4.07
Geraniol	rose, geranium	9.84	69.55	71.83	109.15	131.36	68.48	150.63	42.13
Benzeneacetaldehyde	honey, floral, rose, sweet	13.06 *	4.50	7.97	3.73	2.51	4.51	1.76	2.03
Methyl salicylate	peppermint	2.35	3.87	1.72	4.22	2.07	1.25	1.09	1.03
*p*-Cresol	medicine, phenol, smoke	2.69	1.93	2.18	2.85	2.16	2.82	2.03	5.49 *
Phenylethyl alcohol	honey, spice, rose, lilac	2.57	2.66	4.19	1.69	2.89	10.13 *	1.18	2.39
3-Methyl-butanoic acid	sour, cheesy, disagreeable	<1	<1	<1	<1	2.68	2.13	2.88	4.07
Benzaldehyde	almond, burnt sugar	<1	<1	1.18	<1	<1	<1	<1	<1

^a^: The odor notes were obtained from http://www.flavornet.org/flavornet.html (accessed on 16 January 2019) and http://www.thegoodscentscompany.com/search2.html (accessed on 16 January 2019). * above the data means the OAV of this VFC in a certain sample is significantly higher than that in other samples

## Data Availability

The data presented in this study are available on request from the corresponding author.

## Supplementary Materials

The following supporting information can be downloaded at: https://www.mdpi.com/article/10.3390/foods12071560/s1, Table S1: Sensory evaluation of aroma in black tea samples.; Table S2: Volatile metabolites and relative concentrations detected in black teas by GC-MS.
