# Supplementary material for: Characterization of the Aroma Profiles of Guangdong Black Teas Using Non-Targeted Metabolomics
Source: Foods. 2023 Apr 6;12(7):1560. doi: 10.3390/foods12071560 (PMC10094627; doi:10.3390/foods12071560)
Supplement: Supplementary file 1 [file foods-12-01560-s001.zip › foods-2240337 - Table S1 - for publish.pdf]

Table S1. Sensory evaluation of aroma in black tea samples.

NO.	Sample source		Aroma profile
1	Yingde black tea	YJ-Yingde	Relatively clean and lasting sweet and orchid aroma
2			Slightly clean and lasting sweet aroma, with minty odor
3			Markedly sweet aroma
4			Clean and lasting sweet with orchid aroma
5			Clean and lasting sweet with orchid aroma
6			Relatively clean and lasting sweet and orchid aroma
7			Relatively clean and lasting sweet and orchid aroma
8			Sweet with orchid aroma
9			Relatively clean and lasting sweet and orchid aroma
10			Relatively clean and lasting sweet and orchid aroma
11			Relatively clean and lasting sweet and orchid aroma
12			Relatively clean and lasting sweet and orchid aroma
13		HY-Yingde	Fine, sharp, and lasting sweet and floral aroma
14			Relatively clean and lasting sweet and orchid aroma
15			Fine, sharp and lasting sweet and floral aroma
16	Luokeng black tea		Strong and lasting almond and sweet aroma
17			Relatively clean and lasting sweet and floral odors
18			High and lasting sweet and floral odor
19			Aroma of almond and sweet aroma
20	Renhua black tea		Delicate, lasting medicinal and sweet aroma
21			Delicate, lasting medicinal and sweet aroma
22			Medicinal and sweet floral aroma
23			Medicinal and sweet floral aroma
24			Clean and lasting sweet and floral odor
25			Delicate, lasting medicinal and sweet aroma
26	Meizhou black tea		Relatively clean and lasting sweet and floral odors
27			Medicinal and sweet odors
28			Relatively clean and lasting sweet and floral aroma
29	Heyuan black tea		Relatively pure, strong and lasting honey, sweet and floral aroma
30	Lianshan black tea		Medicinal and sweet aroma
31	Chaozhou black tea		Sweet, floral and medicinal aroma, with some sour odor
